# Exploring mandibular canal variations using CBCT: A study on clinical relevance and risk assessment

**DOI:** 10.6026/973206300214314

**Published:** 2025-12-15

**Authors:** Rahul Srivastava, Hadi Raza, Devina Pradhan, Saquib Khan, Lokesh Sharma, Jahnabi Kakoti

**Affiliations:** 1 Department of Oral Medicine & Radiology, Rama Dental College Hospital & Research Centre, Kanpur, Uttar Pradesh, India; 2 Department of Public Health Dentistry, Clinician Dent-O-Facial Centre, Purnea, Bihar, India; 3 Department of Public Health Dentistry, Rama Dental College Hospital & Research Centre, Kanpur, Uttar Pradesh, India; 4 Department of Oral Medicine & Radiology, Mithila Minorty Dental College, Darbhanga, Bihar, India; 5 Department of Public Health Dentistry, Maharana Pratap Dental College, Kanpur, Uttar Pradesh, India; 6 Clinician, 11 Dental Cares and Aesthetic Centre, Garchuk, Guwahati, Assam, India

**Keywords:** Mandibular canal, CBCT, bifid canals, inferior alveolar nerve, anatomical variations

## Abstract

Anatomical variations of the mandibular canal, including bifid canals and anterior loops, can increase the risk of complications during oral surgical procedures. Traditional radiographic methods often fail to detect these variants accurately, whereas cone-beam computed tomography (CBCT) provides superior visualization. Therefore, it is of interest to describe the prevalence of these variations using CBCT. Hence, a retrospective cross-sectional analysis of 200 CBCT scans (patients aged 18-70 years) was conducted to assess bifid, trifid, retromolar canals, anterior loops and accessory branches in axial, sagittal and coronal planes. Data shows that anatomical variations were present in 29% of cases, with bifid canals most common (18%) followed by anterior loops (11%), indicating that CBCT allows reliable identification of clinically significant mandibular canal variations.

## Background:

The mandibular canal is a crucial anatomical structure within the mandible that transmits the inferior alveolar nerve (IAN), artery and vein from the mandibular foramen to the mental foramen. This neurovascular bundle plays an essential role in innervating and supplying blood to the lower teeth, buccal soft tissues and chin region [1]. Its precise anatomical location and variations are of significant clinical importance, especially in oral and maxillofacial procedures such as implant placement, extraction of impacted third molars, orthognathic surgeries and endodontic interventions [2, 3]. Traditionally, the mandibular canal has been visualized using two-dimensional imaging modalities such as panoramic radiographs. However, these imaging techniques often provide limited information due to distortion, superimposition and magnification errors. This could make it more difficult for them to accurately detect anatomical differences that could raise the risk of intraoperative problems like bifid or trifid canals, anterior loops, or changes in canal course [4]. CBCT is the preferred imaging modality for preoperative examination because it allows for accurate identification of the mandibular canal, its variations and its spatial interaction with other structures [5, 6]. Depending on the age group, imaging modality, classification criteria and population under study, the reported prevalence of BMC varies greatly, ranging from 0.08% to 64.8% [7]. If these differences are not identified before to surgery, they may result in anesthetic failure during inferior alveolar nerve blocks or unintentional nerve damage during surgery [8]. A higher frequency of these anatomical variations in particular populations and ethnic groups has also been observed in a number of studies, indicating a genetic or developmental influence. In order to prevent nerve damage, it is also crucial to take into account changes in the canal's vertical position in respect to the molar roots or its location in relation to the buccal and lingual cortical plates when placing implants [9]. Therefore, it is of interest to report the use of CBCT scans to assess the anatomical variability of the mandibular canal in a specific population, including bifurcation, loop creation and vertical location.

## Materials and Methods:

This retrospective cross-sectional observational study was carried out at Rama Dental College Hospital and Research Centre Mandhana Kanpur in the Department of Oral Medicine and Radiology. Assessing mandibular canal anatomical differences using CBCT images was the main goal. According to the Declaration of Helsinki's ethical criteria, the study was carried out with ethical clearance from the Institutional Ethical Committee (RDCHRC/ETHICSCOMMITTEE/2025/0234 dated 14/03/25). All CBCT scans used were archived records, ensuring no additional radiation exposure was imposed on the subjects.

The following formula is used to calculate the size of the required sample,

n = (z)^2^ p ( 1 - p ) / d^2^

Assuming a prevalence (p) of 20% based on previous literature, a confidence level of 95% (Z = 1.96) and an absolute precision (d) of 5.5%, the estimated sample size was calculated to be 200.A total of 200 CBCT scans were included in the study, selected randomly from the departmental database. The scans were taken between 18/3/2025 and 17/6/2025 and represented adult patients aged between 18 and 70 years. Inclusion criteria required high-quality CBCT scans with fully erupted mandibular permanent teeth, clearly depicting the mandibular canal. Scans showing pathology, previous mandibular surgery, or poor image quality due to motion artifacts or distortion were excluded. This selection helped ensure reliable evaluation of anatomical features. All CBCT scans were acquired using a standardized imaging protocol with CBCT Machine (Carestream CS 8300/3D). The imaging parameters included a field of view ranging from 4 x 4 cm to 12 x 10 cm, tube voltage between 60-90 kVp and a tube current of 2 to 10 mA, with an average scan time of 10-15 seconds. Scans were evaluated usingCBCT Software CS imaging 8.0.25 software and images were viewed in axial, sagittal and coronal planes under standardized dim lighting on a high-resolution diagnostic monitor to enhance visualization accuracy. Each CBCT scan was independently evaluated by two experienced oral radiologists. Anterior variations such as bifid or trifid mandibular canals, retromolar canals, accessory canals and the presence of an anterior loop of the mental nerve were identified by examining the mandibular canal's course, whether it was straight, curved, or serpentine. Additionally, the location of the mandibular canal was assessed in relation to the roots of mandibular molars and its proximity to the buccal and lingual cortical plates. Variations were documented as either unilateral or bilateral. In cases of disagreement between observers, a final consensus was reached through joint discussion. The reliability of observations was measured using Cohen's Kappa coefficient, with a value above 0.8 considered to indicate excellent agreement. All data were systematically entered into Microsoft Excel and analyzed using IBM SPSS Statistics version 25.0 (IBM Corp., Armonk, NY, USA). Descriptive statistics such as mean, standard deviation and frequency distributions were used to summarize the data. The Chi-square test was applied to assess associations between mandibular canal variations and demographic factors like age, gender and laterality. Additionally, independent t-tests and ANOVA were used where necessary to compare canal dimensions across subgroups. A p-value of less than 0.05 was considered statistically significant for all tests performed.

## Results:

Table 1 (see PDF) shows the overall distribution of different anatomical variations. A normal single canal was seen in 71% of cases. The bifid mandibular canal was the most common variation observed, found in 18% of patients, followed by the anterior loop of the mental nerve (11%). Table 2 (see PDF) shows the Laterality of Bifid Mandibular Canal. Among 36 cases of bifid canals, 75% were unilateral, with only 25% presenting bilaterally. Unilateral bifid canals are more common and often missed if bilateral symmetry is assumed during treatment planning. CBCT should be used even for one-sided procedures to avoid overlooking a variation. Table 3 (see PDF) shows the gender-wise distribution of canal variations. Slightly more males showed anatomical variations than females, especially bifid canals. However, the differences were not statistically significant (p > 0.05), indicating that gender does not have a strong influence on the prevalence of these variations. Table 4 (see PDF) shows the age-wise prevalence of anatomical variations. Variation prevalence is relatively consistent across age groups, with a slightly lower percentage in older individuals (25%). The differences were not statistically significant (p = 0.781).

## Discussion:

The current study used cone-beam computed tomography (CBCT) to assess the anatomical differences of the mandibular canal in a sample of 200 people.The results revealed that 29% of the population exhibited anatomical variations, with the bifid mandibular canal (BMC) being the most frequently observed variant (18%), followed by the anterior loop of the mental nerve (11%), retromolar canals (4.5%) and accessory canals (3%). These findings reinforce the increasing awareness that the mandibular canal is not always a single, straight anatomical entity. The prevalence of BMCs in this study is comparable to that reported by Kuribayashi *et al.* who found a prevalence of 15.6% using CBCT among a Japanese population [6]. Similarly, Naitoh *et al.* reported a prevalence of 21% in their CBCT-based study, also among Japanese patients [7]. In contrast, studies using panoramic radiographs report much lower values, typically less than 1%, due to limitations in two-dimensional imaging such as superimposition and lack of depth perception [10, 11]. This highlights the superiority of CBCT in detecting such fine anatomical details. The unilateral presentation of BMCs in 75% of the cases aligns with the observations of Sanchis *et al.* who also found unilateral bifid canals to be more common than bilateral ones [8]. Although variations were more frequent in males (30.4%) than in females (27.3%), the difference was not statistically significant, which agrees with findings from studies such as Soman *et al.* where gender did not significantly affect the occurrence of canal variations [12]. In 11% of instances, the anterior loop of the mental nerve was seen, which is in line with the 10-28% range noted in a prior CBCT study [3]. This variation is clinically important in implant planning in the premolar and mental foramen regions, as unrecognized loops may result in nerve injury or altered sensation if implants are placed too close. Age-wise distribution in the present study did not show any significant trend, suggesting that these variations are likely developmental rather than acquired. Oliveira-Santos *et al.* also found no significant association between age and the presence of mandibular canal variations, which is consistent with this observation [9]. From a clinical standpoint, these findings underscore the importance of pre-surgical CBCT evaluation, especially for procedures involving the posterior mandible, such as third molar extraction, implant placement and mandibular block anesthesia. If anatomical variants like bifid canals are not detected in advance, they may result in issues like incomplete anesthetic, nerve damage, bleeding, or implant failure. It's interesting to note that the current study also found that 61.5% of people had a straight mandibular canal route, 28% had a curved trajectory and 10.5% had a serpentine or irregular path [13]. These results imply that in order to prevent difficulties during osteotomies or nerve repositioning procedures, it is important to meticulously document not only the existence of variations but also the canal's path and location. It's clear that prevalence rates might vary depending on geographic and cultural variety when compared to research done in other communities. Kim *et al.*, for instance, discovered varying accessory canal rates in a Korean group, indicating potential genetic factors [2]. Thus, in order to customize safe treatment regimens, population-specific data are crucial. All things considered, this work adds to the expanding corpus of research highlighting the intricacy of mandibular canal anatomy and the need of CBCT imaging in routine dentistry and surgical procedures. This study may have limited applicability due to its retrospective design and reliance on CBCT scans from a single institution. The sample excluded children and edentulous individuals. Clinical correlations with surgical outcomes or patient symptoms were not established. Additionally, inter-observer reliability was not statistically analyzed and the study did not assess the direct impact of canal variations on anesthetic effectiveness or surgical complications.

## Conclusion:

Anatomical variants of the mandibular canal, particularly bifid canals and anterior loops, are common, often unilateral and show no significant association with age or gender. Given the variability in the canal's position and course, individualized assessment is essential, as conventional radiographs may fail to detect these differences and increase the risk of complications. Cone-beam computed tomography (CBCT) provides superior visualization and should be routinely used in preoperative planning for procedures involving the posterior mandible.
